# Explaining adults’ mental health help-seeking through the lens of the theory of planned behavior: a scoping review

**DOI:** 10.1186/s13643-022-02034-y

**Published:** 2022-08-09

**Authors:** Claire Adams, Eyal Gringart, Natalie Strobel

**Affiliations:** 1 Present Address: School of Arts and Humanities, Edith Cowan University, 270 Joondalup Drive, Joondalup, Perth, Western Australia 6027 Australia; 2 Kurongkurl Katitjin, Edith Cowan University, 2 Bradford Street, Mount Lawley, Western Australia 6050 Australia

**Keywords:** Attitudes, Behavioral intentions, Help-seeking behavior, Health service utilization, Mental health, Planned behavior, Social norms

## Abstract

**Background:**

Despite evidence-based efficacy, mental health services are underutilized due to low rates of help-seeking, leaving unmet mental health needs a global concern. The Theory of Planned Behavior (TPB) has been applied to understand the help-seeking process and in the development of behavior change interventions. The aim of this scoping review was to map the literature on the TPB as applied to mental health help-seeking in adults aged >18 years.

**Methods:**

This scoping review was conducted based on the methodology presented by Arksey and O’Malley (2005). Six databases (CINAHL, PsycINFO, MEDLINE, ProQuest Health and Medicine, ProQuest Dissertations and Theses, Web of Science) and two grey literature sources (OpenGrey, Google Scholar) were systematically searched in February 2018 and updated in March 2020. Studies that explicitly discussed the TPB in the context of mental health help-seeking were initially selected; only studies that explored formal help-seeking for mental health problems and were published in English were retained. Data were extracted using Microsoft Excel.

**Results:**

Initially, 8898 records were identified. Of these, 49 met the selection criteria and were included: 32 were journal articles and 17 were theses. Forty-three papers reported on non-intervention studies and seven articles reported on TPB-based interventions. Most studies (*n* = 39) identified predictors of help-seeking intentions. Attitudes and perceived behavioral control were significant predictors of intentions in 35 and 34 studies, respectively. Subjective norms were a significant predictor of intentions in 23 studies. Few studies aimed to predict help-seeking behavior (*n* = 8). Intentions and perceived behavioral control were significant predictors of behavior in seven and six studies, respectively. Only six TPB-based interventions were identified, all used digital technology to influence help-seeking, with mixed results.

**Conclusions:**

The present scoping review identified a considerable evidence base on the TPB for predicting mental health help-seeking intentions. Attitudes and perceived behavioral control were frequently found to be significant predictors of help-seeking intentions. Knowledge on the TPB for predicting mental health help-seeking behavior, and on TPB-based interventions, is limited. Thus, the role of the TPB in developing help-seeking interventions remains unclear. Recommendations are presented to address such research gaps and inform policy and practice.

**Supplementary Information:**

The online version contains supplementary material available at 10.1186/s13643-022-02034-y.

## Background

Mental health problems are a major public health concern. Recent prevalence estimates indicate 10.7% of the world’s population live with a mental health disorder, and the number of years lived with a mental health disorder has increased by 13.5% from 2007 to 2017, which is likely to be a reflection of increasing prevalence rates and greater life expectancy [1–3]. Associated with the rising prevalence rates of mental health problems is a high level of unmet mental health needs. Unmet mental health needs not only extend the period in which someone lives with the burden of ill mental health, but are also associated with greater levels of disability, reduced quality of life, and an increased likelihood of suicidal ideation and suicide attempts [4, 5].

Pervasive unmet mental health needs have been reported in prominent and large-scale studies of mental health such as the World Health Organization (WHO) and World Mental Health Surveys and are reflected in low rates of service utilization [6–9]. For example, results of the World Mental Health Surveys conducted across 21 countries revealed only 27.6% of people with anxiety disorders received treatment over the course of one year, and less than one in 10 received adequate treatment [10]. People in low- and middle-income countries were the least likely to receive treatment (13.1%). Furthermore, national data from Australia and Canada indicate a little over half of the people who experienced emotional distress in 2013 and 2016 received treatment; however, many people (37% in Australia and 30% in Canada) reported that they did not want treatment [11].

Individual and social factors, such as perceived need for treatment, poor mental health literacy, and low rates of help-seeking have been shown to influence rates of service utilization. Researchers have noted that many people with mental health problems go undiagnosed and untreated, often due to a lack of knowledge about mental health disorders and their treatments, as well as a lack of help-seeking for mental health problems [12, 13]. This has led to a widely recognized mental health treatment gap, whereby more individuals are diagnosed with mental health problems than those who receive treatment [14, 15]. This treatment gap continues to exist even with the growing evidence base on effective mental health treatments.

The literature indicates that despite the effectiveness of evidence-based mental health treatments, most people do not seek help, or delay seeking help, which has significant personal, social, and economic costs [14, 16]. There are many factors that influence help-seeking including stigma, attitudes, knowledge, financial resources, perceived need, and structural barriers/facilitators [12, 17–19]. Understanding these factors and addressing the disparity between the need for mental health services and their use are critical to understanding and addressing mental health outcomes. Still, most of the research on mental health help-seeking has been exploratory and devoid of a theoretical orientation, which has been identified as a gap in the body of knowledge. A clear theoretical framework would greatly improve our ability to understand, explain, predict, and address maladaptive behaviors such as refraining from seeking professional mental health support.

In recent years, researchers have attempted to address this gap [20–22], and a number of theories have been applied to mental health help-seeking [23]. One such theory is the Theory of Planned Behavior (TPB). The TPB is an extension of Ajzen and Fishbein’s Theory of Reasoned Action (TRA) and suggests people make conscious decisions to act, or not act, based on their attitudes, subjective norms, and perceived behavioral control [24]. The theory posits that attitudes towards a behavior influence a person’s plan or intention to act, and subsequently, the action or behavior itself [25]. Subjective norms, defined as perceived social pressures from significant others to perform a behavior, also influence a person’s intentions and behavior [26]. Perceived behavioral control, which is the perceived opportunities, skills, and resources needed to perform a behavior, can influence behavior directly or indirectly as it affects intentions [24]. The inclusion of perceived behavioral control as an antecedent to intentions and behavior is the main difference between the TPB and the TRA. By considering the role of perceived behavioral control, the TPB includes non-motivational factors that influence behavior, thereby overcoming criticisms of the TRA [26, 27]. Thus, the TPB proposes that attitudes, subjective norms, and perceived behavioral control influence behavioral intentions, which predict behavior [24].

The utility of the TPB to explain factors that contribute to decision-making, and how behavior change occurs, is thought to be particularly useful in understanding mental health issues, the help-seeking process, and developing interventions to facilitate mental health behavior change [28–30]. Whilst there is no commonly accepted definition of professional help-seeking, Cornally and Mccarthy [31] defined help-seeking as “a problem focused, planned behavior, involving interpersonal interaction with a selected health-care professional”. Seeking help from a professional, also known as formal help-seeking, involves seeking help from professional sources who can provide appropriate care [16]. Seeking help from a professional is considered optimal for the receipt of evidence-based treatments [23, 32]. The conceptualization of help-seeking as a planned behavior aligns well with the TPB, as it suggests seeking help involves a conscious or planned decision to seek or not seek support. There is also evidence supporting attitudes and intentions as influential to mental health help-seeking behavior, further substantiating the relevance of the TPB in this area [33]. Applying theories that encompass attitudes and intentions when investigating behavior, such as the TPB, is recommended by scholars and has been a major focus of research across fields [34].

Whilst there is no universally accepted theory or model in the area of mental health help-seeking [23], the body of research applying the TPB to understand mental health help-seeking continues to gain momentum, and the TPB has been favored as the most common approach to frame help-seeking in the literature [16]. Studies on the TPB have identified predictors of mental health help-seeking intentions as well as behavior. For example, Tomczyk et al. [29] found attitudes, subjective norms, and self-efficacy (a component of perceived behavioral control), significantly influence intentions, and intentions significantly predict behavior, among people with untreated depressive symptoms in Germany. Moreover, Zorrilla et al. [35] explored predictors of help-seeking intentions among young adults in the USA and found attitudes was a strong, significant predictor of help-seeking intention for mental health services, followed by perceived behavioral control and subjective norms. Conversely, Bohon et al. [36] found partial support for the influence of TPB variables on help-seeking, with attitudes and perceived behavioral control significantly predictive of intentions to seek help, but not subjective norms.

There is a need to synthesize what is known about the TPB in the context of mental health help-seeking, to facilitate the identification of factors related to mental health help-seeking, which may provide viable targets for intervention to encourage mental health service utilization and potentially reduce the treatment gap. Recent research has attempted to improve rates of mental health help-seeking through TPB-based interventions [37–39], however these are yet to be reviewed. It is important to understand what efforts have been made to improve mental health help-seeking to progress the effectiveness of interventions and inform future directions.

The aim of the current scoping review was to map the literature on the TPB when applied to mental health help-seeking. Given the favorable results of the TPB in the health field, and logical expansion of the TPB to mental health help-seeking, this theory provides an important evidence base from which mental health help-seeking can be understood and improved. A scoping review allowed the identification of different types and sources of evidence on this topic, as well as gaps in them, which limit our understanding. The purpose of this scoping review was to:

1. Identify how the TPB has been applied to mental health help-seeking
2. Identify and summarize the theoretical factors, which influence help-seeking intentions and behavior
3. Explore the current state of knowledge on TPB-based interventions designed to improve mental health help-seeking
4. Highlight how this understanding can inform future research and practice on mental health help-seeking in adult populations.

## Methods

The current scoping review was based on the methodology outlined by Arksey and O’Malley [40]. Arksey and O’Malley proposed a five-stage process, which includes (1) identifying the research question, (2) identifying relevant studies, (3) study selection, (4) charting the data, (5) collating, summarizing, and reporting the results. This process was followed, and the scoping review is reported based on the Preferred Reporting Items for Systematic reviews and Meta-Analyses extension for Scoping Reviews (PRISMA-ScR) checklist (see Additional file 1) [41].

### Review protocol

The scoping review protocol was drafted using Arksey and O’Malley’s five-stage process and was published on the Open Science Framework on 9th November 2017 (https://osf.io/73c5a/).

### Identifying the research question

The research question for this scoping review was: What is known about the application of the TPB to mental health help-seeking in adults?

### Criteria for study inclusion

Table 1 summarizes the study eligibility criteria.

**Table 1:** Eligibility criteria

Characteristics	Criteria
**Inclusion criteria**	
Study characteristics	
Types of studies	All studies that explicitly discussed the TPB in the context of mental health help-seeking, independently of the TRA
Participants	Adults >18 years
	With and without mental health problems
	No restrictions on sociodemographic characteristics
Concepts	Source of help: primary health care provider or mental health specialist
	Mental health problem: any symptoms of emotional distress or psychological disturbance
Measures	No restrictions
Outcomes	Primary: attitudes, subjective norms, perceived behavioral control, help-seeking intentions
	Secondary: help-seeking behaviors
Setting	No restrictions
Review characteristics	
Years considered	No restrictions
Language	English
Publication status	Published/submitted journal articles and gray literature
**Exclusion criteria**	
Study characteristics	
Types of studies	Editorials, opinion pieces, and conference abstracts
Participants	People with cognitive deficits (e.g., dementia, intellectual disabilities, head injuries)
	People diagnosed with schizophrenia
	Drug users
	Survivors of cancer
Concepts	Source of help: seeking help from self-help programs and resources
	Mental health problem: substance abuse and eating disorders
	Secondary help-seeking (seeking help for someone else)
Measures	No restrictions
Outcomes	Any primary or secondary outcome that was not examined independently of other variables
Setting	No restrictions
Review characteristics	
Years considered	No restrictions
Language	Languages other than English
Publication status	No restrictions

#### Types of studies

All studies, which explicitly discussed the TPB in the context of mental health help-seeking, independently of the TRA, were included in the current review. Studies that utilized mediation, moderation, and extended models of the TPB were included only if they examined all variables in the TPB model, and these variables were examined independently (attitudes, subjective norms, perceived behavioral control, intentions and, where applicable, behavior). For the purposes of this study, we defined traditional TPB models as models which included only the aforementioned TPB variables and extended TPB models as those which included any additional predictor(s).

Intervention studies were included if they utilized the TPB model in the design and development of the intervention. Editorials, opinion pieces, and conference abstracts were excluded. Qualitative studies were also excluded as these tended to be formative research, which use the TPB as an exploratory framework rather than apply the TPB constructs to mental health help-seeking.

#### Participants

This review included papers focused on adults aged >18 years. Samples with and without mental health problems were included, as well as samples restricted to specific sociodemographic criteria (such as age, gender, and occupation). Populations with cognitive deficits such as dementia, intellectual disabilities, and head injuries were excluded, as a person’s cognitive ability directly affects their capacity to seek help as well as process the TPB variables. Studies specific to people diagnosed with schizophrenia, drug users, and survivors of cancer were also excluded due to the cognitive impairments often found in these populations resulting from their treatments or drug use.

#### Concepts

Only papers focused on formal mental health help-seeking were included in the current review. Formal help-seeking has been defined as seeking help from professional sources who can provide appropriate care [16]. In the context of mental health help-seeking, a professional source may include a primary health care provider (general practitioner, nurse, medical specialist) or mental health specialist (psychologist, social worker, counselor, psychiatrist). Formal mental health help-seeking may be face-to-face or online (e.g., e-counseling) as long as it was from a professional source. Self-help programs and resources were excluded, and papers which did not distinguish between formal and informal sources of support were also excluded.

Studies were included if the reason for seeking help was for mental health problems or concerns. For the purposes of the current review, a mental health problem included any symptoms of emotional distress or psychological disturbance, to ensure all studies relevant to mental health were encompassed. The mental health problem did not have to be formally diagnosed as a mental disorder or meet established criteria for a mental disorder.

Some mental health problems such as substance abuse and eating disorders have been considered health-related behaviors in previous research [42, 43]. In the present review, these conditions were excluded, as our aim was to examine how the TPB has been applied to mental health problems beyond the traditional application of the TPB to health-related behaviors.

Additionally, studies which focused on secondary help-seeking (seeking help for someone else such as a peer, family member, or friend) were excluded as we sought self-agency over help-seeking behavior, in line with the TPB assertion that behavior must be under volitional control [24].

#### Measures

Standardized quantitative measures of the TPB are scarce, and therefore, all questionnaires and tools that measure TPB variables were included.

#### Outcomes

The primary outcomes founded in the TPB framework were (1) attitudes: attitudes toward seeking help for mental health problems; (2) subjective norms: beliefs about whether others approve or disapprove of help-seeking; (3) perceived behavioral control: beliefs about one’s own ability to seek help for mental health problems; and (4) help-seeking intentions: conscious decision to seek or not seek help.

The secondary outcome was help-seeking behaviors, defined as actual help-seeking from a health professional.

### Identifying relevant studies

Searches were conducted across six databases (CINAHL, PsycINFO, MEDLINE, ProQuest Health and Medicine, ProQuest Dissertations and Theses, and Web of Science) and two gray literature sources (OpenGrey and Google Scholar). Only papers published in English were included. The searches were run during February 2018 and updated in March 2020. With the help of an experienced subject librarian, keywords were selected, which allowed for breadth of coverage (see Table 2).

**Table 2:** Search strategy

Keywords	
(theory of planned behav*) OR (Ajzen) AND ((psych* OR mental*) AND (ill* OR disorder* OR symptom* OR disease* OR health*))	

The above search strategy was used for each database search, and keywords from this search strategy were used to search the gray literature sources. Multiple combinations of the keywords were used to conduct the Google Scholar searches, and the first 50 pages (representing 1000 results) were screened using Google search engines relevancy ranking to order the results [44]. Reference list searches were also conducted from articles selected for inclusion to identify other relevant studies, which may have been absent in the electronic searches.

### Study selection

All titles and abstracts retrieved through the literature searches were reviewed independently by two researchers to identify studies that met the inclusion criteria. The reviewers met during the title and abstract screening and at the conclusion of this stage to discuss and resolve conflicts regarding the inclusion of articles. The inclusion criteria at the title and abstract level were limited to any paper that utilized the TPB or any of its primary variables (attitudes, subjective norms, perceived behavioral control) in relation to mental health problems. Studies excluded at this stage were those including children under the age of 18 exclusively, people with cognitive deficits, and studies not published in English. The covidence systematic review platform was used for title and abstract screening [45].

Once the relevant studies had been selected, the full-text was retrieved and read independently by two researchers to determine eligibility for inclusion, based on the criteria described above. A third senior reviewer was consulted to resolve conflicts regarding the inclusion of articles, and a final decision was reached once a consensus was achieved.

### Charting the data

A standard data charting form was developed and pilot-tested in Microsoft Excel before data extraction. Data collected from each study were organized according to key information: basic descriptors (title, first author, year of publication, publication type, country), eligibility information (aim/purpose of the paper, reason for seeking help, source of help), study population (population group, mental health status, sample size, setting, method of recruitment, sociodemographic information), research methods (study design, measures used, analysis, intervention details if applicable), primary and secondary outcome measures (attitudes, subjective norms, perceived behavioral control, intentions, behavior), and description of key findings.

### Collating, summarizing, and reporting the results

Descriptive tables were used to outline the key information from each paper. Based on the content of the articles and the present research question, the studies were organized according to non-interventions and interventions. Tables were used to highlight the pertinent information from the non-intervention and intervention studies according to our review objectives.

## Results

**Fig. 1:**
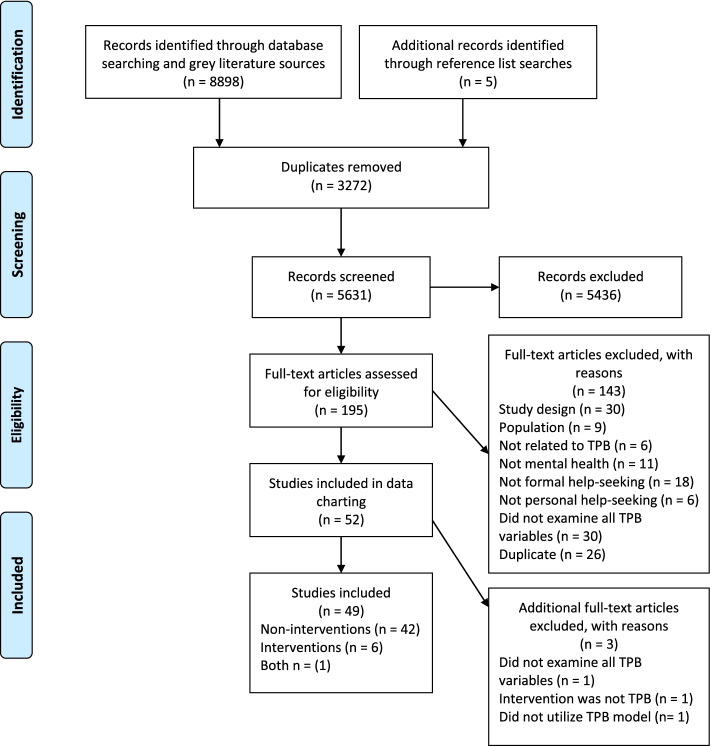
Flow diagram of study selection

A PRISMA flow diagram is presented in Fig. 1, which outlines the process of selecting studies. There were 8898 records identified through the literature searches. After the removal of duplicates, 5626 records were screened for eligibility. There were 5436 records excluded at the title and abstract stage, 143 articles excluded after full-text screening and three articles excluded after data extraction. The reasons for exclusion are provided in Fig. 1. A further 5 articles were identified through reference list searches and screened. This process resulted in the inclusion of 49 articles.

### Characteristics of studies

**Fig. 2:**
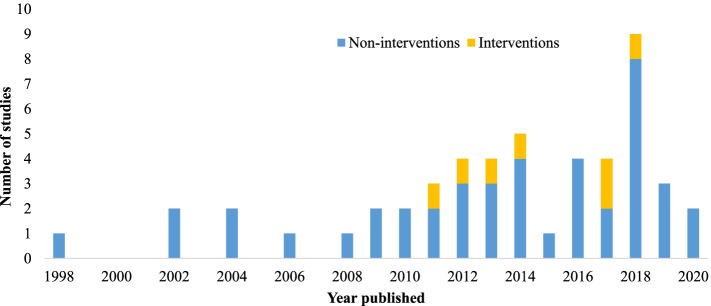
Stacked histogram of the number of studies published per year

The 49 articles that were chosen were published between 1998 and 2020; 32 (65%) were journal articles and 17 (35%) were theses. Of these, 42 (86%) were non-intervention studies, six (12%) were intervention studies, and one paper (2%) reported on both cross-sectional data and an intervention (Fig. 2).

Most studies were conducted in North America (*n* = 27, 55%), followed by Europe (*n* = 9, 19%), Asia (*n* = 8, 16%), Oceania (*n* = 4, 8%), and Africa (*n* = 1, 2%). In terms of the populations, most targeted college students (*n* studies = 16, 33%; *n* participants = 6048), followed by community samples (*n* studies = 12, 25%; *n* participants = 7305), both students and community samples (*n* studies = 1, 2%; *n* students = 320; *n* community adults = 208), armed forces or emergency service workers (*n* studies = 8, 16%; *n* participants = 1910), older adults (*n* studies = 2, 4%; *n* participants = 568), and other specific populations (*n* studies = 10, 20%; *n* participants = 3137).

Most studies included all participants with and without symptoms of mental health problems (*n* studies = 42, 86%; *n* participants = 17371), three studies (6%; *n* participants = 1542) only included participants with psychological symptomatology, two studies (4%; *n* participants = 160) targeted people who screened positive for a mental disorder, one study (2%; *n* participants = 400) included dysphoric individuals only, and an intervention design paper (*n* studies = 1, 2%; *n* participants = 23) only included young adults with experience in seeking help for mental health issues.

### TPB-based non-intervention studies

Forty-three articles reported on non-intervention studies [21, 29, 35, 36, 46–83]. Of these, 28 (65%) were journal articles and 15 (35%) were theses. A summary of the final selected non-intervention articles is given in Additional file 2, Supplementary Table 1.

Two journal articles reported on the same sample [77, 79], and one thesis reported pre-intervention cross-sectional data and intervention data and thus was included in both sections [58]. One study reported on post-intervention data only and was included under non-intervention studies as the intervention was not based on the TPB [62]. The results of the study are not reported due to the study design.

Most of the non-intervention studies were conducted in North America (*n* = 23, 53%), followed by Europe (*n* = 9, 21%), Asia (*n* = 8, 19%), Oceania (*n* = 2, 5%), and Africa (*n* studies = 1, 2%). The most commonly targeted populations were students (*n* studies = 14, 33%; *n* participants = 5588), followed by armed forces or emergency service workers (*n* studies = 7, 16%; *n* participants = 1900) and community samples (*n* studies = 10, 23%; *n* participants = 7231).

Of the 43 non-intervention papers, 38 were cross-sectional studies that aimed to identify predictors of mental health help-seeking intentions and/or behaviors. Five of the cross-sectional papers included a psychometric study or elicitation study, which designed and/or evaluated measures to assess TPB variables, for use in the main cross-sectional study [36, 50, 53, 54, 59]. Three papers were solely psychometric evaluations, which examined the validity and reliability of attitudinal mental health help-seeking scales. Two centered on the reliability and validity of the Inventory of Attitudes towards Seeking Mental Health Services (IASMHS) [49, 84]. The third paper developed and tested the Mental Help-Seeking Attitudes Scale (MHSAS) [76]. One psychometric study also reported on predictors of help-seeking intentions [84].

In addition, one paper was an experimental between-group design, which aimed to influence intentions to seek help for depression through evoking positive emotions using nostalgic messages [62], and one paper presented baseline data from a randomized controlled trial examining students’ intentions to seek help and actual counselling usage [58].

The type of mental health problem was rarely defined in the literature, most commonly referred to as a psychological problem or mental health problem/concern (*n* studies = 21, 49%). Other terms used included psychological stress/distress, personal and/or emotional problems, psychological difficulties, mental health conditions, persistent issues, and common concerns. Some studies gave examples of mental health problems, for example, Mo and Mak [21] used the term mental health problems and provided a definition to include low mood, tense, anxious, and problems sleeping. Stecker et al. [55] used the term mental health concerns and defined these to include major depressive disorder, panic disorder, generalized anxiety, post-traumatic stress disorder, and/or alcohol abuse disorder. Some studies presented a list of symptoms or problems such as depressed mood, anxiety, relationship difficulties, personal concerns, memory loss, and alcohol or drug abuse [50, 51, 59].

Depression was the only mental health problem to be examined independently, with eight studies (19%) focusing only on help-seeking for depression, and one study (2%) focusing on depression and suicidal thoughts [35, 36, 48, 53, 58, 60, 62, 72]. These studies included all participants irrespective of their mental health status, with the exception of one paper which included only dysphoric individuals [60]. Additionally, three studies (7%) explored help-seeking for suicidality as well as other emotional or mental health problems [54, 66, 80].

As only formal mental health help-seeking was included, most studies reported the source of help as mental health services, psychological/psychiatric help, mental health professional, or a combination of these terms (*n* studies = 27, 63%). Additional terminology included counseling (*n* studies = 7, 17%), treatment (*n* studies = 5, 12%), cyber-counseling (*n* studies = 1, 2%), therapy for depression (*n* studies = 1, 2%), psychotherapy (*n* studies = 1, 2%), and prison psychologist (*n* studies = 1, 2%). Of the studies that defined the type of service, common examples included social workers, counselors, psychologists, and psychiatrists. Six studies (14%) also specified physicians/general practitioners within their definition [49, 55, 57, 67, 71, 75].

#### Factors which influence help-seeking intentions and behavior

The theoretical factors, which predict help-seeking intentions and behavior, are summarized in Tables 3 and 4, respectively. Some studies (*n* = 13, 33%) used traditional models of the TPB to explore help-seeking, whereby only the original TPB variables (attitudes, subjective norms, perceived behavioral control, intentions, and where applicable, behavior) were included in the model. Other studies (*n* = 19, 49%) used extended models of the TPB, in which the original TPB variables and additional variables were included in the model (either as predictors or control variables), to identify further predictors of mental health help-seeking relevant to the population. Seven studies (18%) explored both traditional and extended models.

**Table 3:** Direct predictors of mental health help-seeking intentions

First author (year)	Attitudes	Subjective norms	Perceived behavioral control
Traditional models			
Aldalaykeh (2019) [46]	✓	✓	✓
Bohon (2016) [36]	✓	✗	✓
Chandrasekara (2016) [73]	✓	✓	✓
Chang (2012) [58]	✗	✓^e^	✓
Clansy (1998) [59]	✓	✗	✗
Damghanian (2018) [75]	✓	✓	✓
Hess (2013) [78]	✓	✓	✓
Hyland (2012a) [77]	✓	✓	✓
Jarvis (2002) [63]	✓	✗	✓
Kgathi (2011) [81]	✓	✓	✓
Lee (2016b) [82]	✗	✗	✓^a^
Lee (2016b) using IASMHS [82]	✓	✗	✓
Miller (2004) [65]	✓	✓	✓
Mo (2009) [21]	✓	✓	✓
Pumpuang (2018) [52]	✓	✓	✗
Rathbone (2014) [68]	✓	✗	✓
Schomerus (2009) [53]	✓	✓	✓^c^
Stecker (2010) [55]	✓	✗	✓
Taylor (2018) [70]	✓	✗	✓
Tomczyk (2020) [29]	✓	✓	✓^a^
Westerhof (2008) [57]	✓	✗	✓
Extended models			
Aldalaykeh (2019) [46]	✓	✓	✓
Chang (2012) [58]	✗	✗	✓
Cuyler (2019) [74]	✓	✗	✗
Damghanian (2018) [75]	✓	✓	✓
Farmer (2013) [60]	✗	✓	✓^f^
Hyland (2015) [84]	✓	✗	✓
Hyland (2012b) [79]	✗	✓	✓^a^
Karras (2018) [80]	✓^a^	✓^ad^	✓^a^
Lee (2016a) [64]	✓	✓	✓^a^
Li (2017) [47]	✓	✓	✗
Li (2018) [83]	✓	✗	✗
Logsdon (2018) [48]	✓	✗	✓^e^
Mak (2014) [50]	✓	✓	✓
Mesidor (2014) [51]	✗	✗	✓
Mills (2010)^g^ [66]	✓	✗	✗
Mo (2009) [21]	✓	✓	✓
O’Flaherty (2017) [67]	✓	✗	✓
Rathbone (2014) [68]	✓	✗	✓
Seyala (2011) [69]	✓	✓^c^	✓
Skogstad (2006) [54]	✓	✓	✓
Taylor (2018) [70]	✓	✗	✓
Teo (2020) [56]	✓	✓	✓
Walther (2002) [71]	✓^b^	✓^eb^	✓^b^
Westerhof (2008) [57]	✓	✗	✓
Woods (2013) [72]	✓	✓	✗
Woods (2013) mediation models only [72]	✓	✓	✓
Zorrilla (2019) [35]	✓	✓	✓

^a^Only specific component(s) of this variable were significant

^b^Help-seeking from a mental health professional only

^c^Not significant in a smaller comparison sample

^d^Significant for suicidal thoughts and behaviors only

^e^Inverse association with intentions

^f^Significant in most models

^g^Taken from the results table not the abstract which reports different findings

**Table 4:** Direct predictors of mental health help-seeking behavior

First author (year)	Perceived behavioral control	Intentions
Traditional models		
Chandrasekara (2016) [73]	✓	✓
Chang (2012) [58]	✓	✗
Damghanian (2018) [75]	✓	✓
Lee (2016b) [82]	✗^a^	✓
Lee (2016b) using IASMHS [82]	✓	✓
Li (2017) [47]	✓	✓
Li (2018) [83]	✓	✓
Tomczyk (2020) [29]	✗	✓
Extended models		
Chang (2012) [58]	✓	✗
Damghanian (2018) [75]	✓	✓
Li (2017) [47]	✗	✓^b^
Li (2018) [83]	✗	✓
Stecker (2010) [55]	✗^a^	✓

^a^Only measured control factors

^b^Significant in most models

Overall, 39 studies identified predictors of mental health help-seeking; 31 studies identified predictors of mental health help-seeking intentions only, and eight studies identified predictors of intentions and behavior.

From the 39 studies that identified predictors of mental health help-seeking intentions, attitudes was a significant predictor of intentions in 35 (90%) studies, perceived behavioral control was a significant predictor of intentions in 34 (87%) studies, and subjective norms was a significant predictor of intentions in 23 (59%) studies. The variance in mental health help-seeking intentions explained by the TPB ranged from 7 to 93% for both the traditional and extended models.

There were eight articles that examined factors, which influence mental health help-seeking behavior. Seven of the eight articles (88%) found intentions to be a significant predictor of mental health help-seeking behavior, and six of the eight (75%) articles found perceived behavioral control to be a significant predictor of behavior. Two of the articles (25%), which did not support perceived behavioral control as a predictor of help-seeking behavior only examined one component of this construct, control factors, which may account for the insignificant findings [55, 64]. Furthermore, two articles (25%) found perceived behavioral control to be a significant predictor of behavior in the traditional TPB model, but not when the model was extended to include other variables [47, 83].

An additional paper examined help-seeking behavior but did not utilize the full TPB model [60]. In this paper, intentions were found to be a significant direct predictor of prospective help-seeking, and direct relationships were also found between attitudes and prospective help-seeking, and subjective norms and current help-seeking.

Overall, the variance in mental health help-seeking behavior explained by the TPB ranged from 3 to 61% for the traditional models and 10 to 63% for the extended models.

### TPB-based interventions

Seven articles were identified on TPB-based interventions; four were journal articles and three were theses [30, 37–39, 58, 85, 86]. The seven articles comprised six unique interventions, which used the TPB model to improve intentions or behavior to seek professional help. A summary of the final selected intervention articles is given in Additional file 2, Supplementary Table 2.

Three of the interventions were evaluated in randomized controlled trials, two were evaluated in pre-post studies, and one was assessed using a randomized post-test-only design. All six interventions used digital technology: two interventions were short videos on seeking counseling services, three were web-based tools, and one was a theory-guided multimedia presentation. The target population were mainly young adults (*n* studies = 5, *n* participants = 704), one study targeted USA war veterans (*n* participants = 10), and one study focused on adolescent mothers with a mean age of 18 (*n* participants = 289).

The findings from each study were mixed. One study demonstrated improvements in mental health help-seeking intentions and behavior after intervention. Logsdon et al. [39] employed a pre-post design to evaluate an Internet-based depression intervention encouraging adolescent mothers to seek depression treatment and found a statistically significant increase in both intentions to seek treatment and actually receiving treatment over time in the intervention group compared to the control group. Another study that designed a web-based psychoeducational tool for USA veterans reported an increase in willingness to consider mental health treatment post-intervention [30].

Two studies, both using video interventions on attending counseling, demonstrated significant improvements in all three TPB predictor variables (attitudes, subjective norms, and perceived behavioral control) immediately after the intervention [58, 85]. Chang [58] also conducted a follow-up and found sustained improvements in subjective norms and perceived behavioral control at 4-weeks post-intervention. In both studies, changes in predictor variables did not translate to improvements in intentions or behavior. Furthermore, Lindsley’s [86] guided multimedia presentation on treatment seeking reported findings consistent with overall improvements in TPB predictor variables after intervention; however, this declined over time and no differences between the experimental and control group were found. Finally, an online navigation tool for young adults designed to match participants with appropriate services did not demonstrate improvements in help-seeking intentions; changes in other TPB variables were not assessed [37, 38].

## Discussion

The current scoping review mapped existing literature on the TPB when applied to mental health help-seeking. Of the 49 articles included, the earliest paper identified was published in 1998, with a rise in the number of studies since 2008, indicating growing interest in this field. Most studies were conducted in western countries (North America and Europe), with college students and community samples the most common populations targeted. Participants with and without mental health problems were typically included, which aligns with an early intervention approach to mental health care [87].

This body of research typically applied the TPB to predict mental health help-seeking intentions (*n* = 39). Most studies found the TPB accounted for a large amount of variance in intentions; however, the range in variance explained was large. Of the theoretical factors, which influence mental health help-seeking intentions, attitudes and perceived behavioral control were frequently found to be significant predictors of help-seeking intentions, and subjective norms were found to be a significant predictor in more than half of the studies. This indicates that across population groups, a person’s favorable or unfavorable evaluation towards using mental health-related services, and their perceived resources, skills, and opportunities to access services, influence their intentions to seek help. For example, the belief that seeing a mental health professional will be helpful, and beliefs regarding potential costs of mental health consultations, influence help-seeking intentions [53]. The influence of subjective norms on intentions, that is, perceived social pressures to seek help, was less consistent, which may be due to differences in cultural norms and beliefs. For example, western cultures are typically individualistic and therefore, people from these cultures may be more motivated to act according to their own goals and beliefs [88]. In the present review, we found subjective norms were not statistically significant predictors of help-seeking intentions in some studies conducted in the USA [36, 55]. In studies conducted in non-western cultures such as South Asia and among Chinese populations, social pressures were found to significantly predict help-seeking intentions [21, 73]. This may be reflective of the collectivistic nature of these societies, which traditionally encourage interconnectedness with others [88].

Indeed, cultural factors such as race, ethnicity, and religion have been shown to influence help-seeking intentions and behavior. For example, mental health problems are commonly stigmatized in Chinese societies, which hinders people’s intentions to seek help for fear of social rejection [50, 64]. In some extended and modified models of the TPB, variables such as adherence to Asian values, shame/izzat, and a Strong Black Woman ideal were included and were shown to significantly predict help-seeking intentions [22, 47, 72]. Some authors have advocated for the use of extended models of the TPB to better explain help-seeking intentions and behavior [89, 90]. In the present scoping review, most non-intervention studies (67%) used extended models or both traditional and extended models to conceptualize help-seeking intentions; however, the variances explained by these models were comparable to traditional TPB models. Thus, factors that influence help-seeking intentions beyond the theoretical factors in the TPB require further investigation.

Fewer studies applied the TPB to predict mental health help-seeking behavior (*n* = 8). Intentions and perceived behavioral control were significant predictors of behavior in most of these studies, supporting the TPB framework. Of the studies that did predict help-seeking behavior, past and/or present behaviors were commonly used as the outcome. We only located three studies, which explored the ability of the TPB to predict future behavior [29, 55, 75]. This is a key gap in the evidence related to predicting whether people would seek help for mental health problems if a future need arose. Whilst studies have found past behavior to have been a good indicator of future behavior, this is not always the case [24]. Additionally, there is a widely recognized intention–behavior gap, where positive intentions are not always antecedent for actual behavior [91]. Thus, there exists an important opportunity to develop TPB models for predicting future behavior, to identify groups who are in need and yet are less likely than other populations to seek help, and to enable targeted interventions to address their needs as well as increase help-seeking behaviors among them.

The current state of knowledge on TPB-based interventions for mental health help-seeking is in its infancy, with few studies published, all within the last decade. It is both difficult and premature to draw conclusions as to the role of the TPB as a framework for developing behavior change interventions for mental health help-seeking, due to the variation in predictor variables examined, lack of diversity in populations, and limited ways in which the interventions were delivered. Of the seven studies we located on TPB-based interventions to improve mental health help-seeking, six used digital technology, mostly to target young adults. This is of concern, given recommendations from researchers to use the TPB to promote behavioral change across populations, and the various approaches which can be trialed to facilitate the greatest change in behavior, e.g., persuasive communications, face-to-face discussions, and observational modeling [42, 92, 93]. There is a need and room for the development and evaluation of TPB-based interventions using different modes of delivery and targeting both community samples and at-risk populations, which may be effective in promoting help-seeking and facilitating service utilization.

There is also space for the TPB to be applied across subgroups who experience disparities in service provision, to explain intentions and behavior towards mental health service utilization; however, this has been largely overlooked in studies to date. Background factors such as age, race, ethnicity, and social class are associated with different behavioral beliefs, behavioral intentions, and prevalence rates of behaviors [94]. Such factors can be incorporated into the TPB model to identify inequities in health and health-related behaviors and guide the allocation of resources to better meet the needs of diverse communities who have differential access to care [94]. For example, people from culturally and linguistically diverse groups are less likely to engage mental health services than members of the wider population, due to a lack of knowledge of services and how to access them, communication barriers, financial barriers, racism, and culturally insensitive practices [95–97]. Moreover, attitudes towards and beliefs about mental health problems and treatments among diverse groups may differ from dominant Western approaches, and access to culturally appropriate care is often limited [98]. The TPB can be applied to identify background factors that influence help-seeking, and attitudes, norms, and control beliefs associated with help-seeking among racial and ethnic minority groups, providing insight into service access and use. Such research is important to accurately address the needs of marginalized populations and promote inclusive practices.

### Implications for future research and practice

**Fig. 3:**
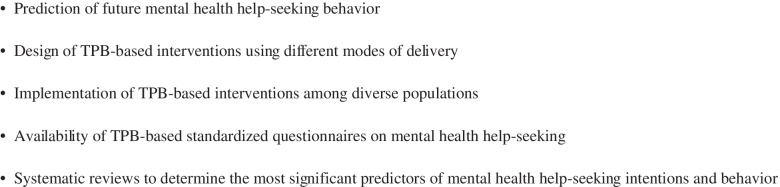
Research gaps

The current scoping review provides a resource for researchers and practitioners to understand mental health help-seeking through the lens of TPB. Prominent research gaps are highlighted in Fig. 3.

There is scope for a number of systematic reviews to determine the most significant predictors of mental health help-seeking intentions and behavior, which can be used as target areas for intervention. Systematic reviewers may wish to focus their investigations on determining the key TPB-based predictors of mental health help-seeking among student samples, given the predominance of studies on this population. There is also scope to conduct a systematic review on methods for measuring TPB variables due to the diversity in quantitative tools used to assess these constructs. This will enable more accurate measurement of these variables and greater ability to compare findings. An investigation of the most reliable evidence for the effects of TPB-based interventions on mental health help-seeking intentions and behaviors is also warranted. Such reviews could provide stakeholders a sound basis for their decisions on how to assess and improve mental health help-seeking.

### Strengths and limitations

By employing a scoping review methodology and a systematic approach to the selection of studies, we conducted a comprehensive and structured review of the literature in line with current recommendations for good practice [99]. This clear structure makes the current results more accessible to stakeholders interested in applying the findings to inform policy and practice [40]. It is still possible that we did not locate all relevant studies or misclassified relevant papers.

Whilst we aimed to include only those studies relevant to adults, defined as people aged 18 years and over, there were three studies which did not define the age of participants [65, 66, 68]. As the sample in Miller [65] comprised lawyers, it is safe to assume that the age of participants was >18 years. In Rathbone (2014) and Mills (2010), however, the target samples were college students, and it is, therefore, possible that the mean age of participants in these studies was <18 years [66, 68]. As we cannot be certain, these three studies were included in this review to ensure we did not falsely exclude relevant papers.

A further limitation is the paucity of TPB-based standardized questionnaires on mental health help-seeking, which restricts our confidence in the accurate measurement of TPB variables. Many authors have created their own measures or adapted previous measures to fit their study contexts [21, 53, 78]. Additionally, validated tools such as the Attitude Toward Seeking Professional Psychological Help Scale (ATSPPHS) and IASMHS have been constructed and applied to mental health help-seeking, but do not necessarily reflect the true nature of the TPB. For example, the ATSPPHS has been widely used as a measure of help-seeking attitudes; however, this tool precedes the TPB and its development was not guided by any explicit theoretical framework [49, 76]. The IASMHS was designed to improve upon this measure by including items, which directly target the TPB predictor variables; however, the three subscales of the IASMHS only loosely reflect the TPB and contain other constructs such as mental health literacy, self-disclosure, avoidance coping, or self-concept clarity [49, 76]. We, therefore, cannot detect whether intentions to seek help were influenced by the components of the TPB or the overlapping or related constructs inherent in this measure. Furthermore, studies that used the IASMHS but did not reference the TPB would not have been identified by our searches but may add to our understanding of mental health help-seeking within a TPB framework. More consistency is needed in the assessment of TPB variables to strengthen the conclusions drawn about the explanatory role of the TPB in the present context.

Additionally, we applied a strict inclusion and exclusion criteria, which may have resulted in the exclusion of closely related studies. We chose this approach to strengthen our understanding of the original TPB framework, without modifications.

## Conclusions

As the TPB is one of the most prominent theories in the social and behavioral sciences, the current scoping review was prudent to improve our understanding of the role of the TPB in explaining mental health help-seeking among adults. With the increase in effective psychological treatments, there is a pressing need for researchers and clinicians to promote engagement with mental health services to alleviate some of the personal and societal costs associated with ill mental health.

As a result of mapping the evidence base, we have identified a considerable body of research on the TPB for predicting mental health help-seeking intentions. Attitudes and perceived behavioral control, in particular, appear to be well-cited factors influencing people’s intentions to seek or not seek help. Less attention was paid to help-seeking behavior; however, among the eight studies that explored help-seeking behavior, intentions and perceived behavioral control were found to be significant predictors of help-seeking behavior, which further supports the TPB in this context. The evidence base for TPB-based interventions to improve mental health help-seeking is in its early stages, and there appears to be a number of gaps in the literature that limit our understanding of the applicability of the TPB in this context and warrant further research.

An important line of future enquiry could be to utilize the theoretical framework of the TPB to find ways to increase help-seeking behavior to improve mental health outcomes. A particular focus should be paid to groups at-risk of mental health problems, such as veterans, people with chronic illnesses, and immigrants, to reduce the personal and societal burden of unmet mental health needs. The current scoping review provides a broad map of the evidence related to mental health help-seeking, which can inform future research, policy, and practice.

## Supplementary Information

**Additional file 1.** Preferred Reporting Items for Systematic reviews and Meta-Analyses extension for Scoping Reviews (PRISMA-ScR) Checklist.

**Additional file 2: Table S1**. Characteristics of studies – non-interventions. **Table S2**. Characteristics of studies – interventions.

## Data Availability

All data generated or analyzed during this study are included in this published article [and its supplementary information files].

## Abbreviations

ATSPPHS: Attitude Toward Seeking Professional Psychological Help Scale
IASMHS: Inventory of Attitudes towards Seeking Mental Health Services
MHSAS: Mental Help-Seeking Attitudes Scale
PRISMA-ScR: Preferred Reporting Items for Systematic reviews and Meta-Analyses extension for Scoping Reviews
TPB: Theory of Planned Behavior
TRA: Theory of Reasoned Action
WHO: World Health Organization
