# Supplementary material for: Explaining adults’ mental health help-seeking through the lens of the theory of planned behavior: a scoping review
Source: Syst Rev. 2022 Aug 9;11:160. doi: 10.1186/s13643-022-02034-y (PMC9361557; doi:10.1186/s13643-022-02034-y)
Supplement: Supplementary file 2 — Additional file 2: Table S1. Characteristics of studies – non-interventions. Table S2. Characteristics of studies – interventions. [file 13643_2022_2034_MOESM2_ESM.docx]

**Additional file 2**

| **Supplementary Table 1** Characteristics of studies – non-interventions | | | | | |
| --- | --- | --- | --- | --- | --- |
| First author (year); country | Participants | Aim of study | Study design | Type of mental health problem(s) | Source of help |
| Journal articles |  |  |  |  |  |
| 1. Aldalaykeh (2019); Jordan | Students N = 134 M age = 20 years Range = not reported 37% male | The purpose of this study is to a) investigate the effect of attitude toward mental health services, subjective norm, and perceived behavioral control on subjects’ intention to seek help of mental health services | Cross-sectional | Psychological stress | Mental health services |
| 2. Bohon (2016); USA | Students Study 1 N = 279 M age = 21 years Range = 18 to 57 years 27% male Study 2 N = 71 Study 3 N = 495 M age = 21 years Range = 17 to 57 years 29% male | To study the usefulness of using the TPB to predict intention to seek mental health services and to investigate barriers to care-seeking behavior. | Study 1 and 2: psychometric studies Study 3: cross-sectional study from larger randomized controlled trial (RCT) | Depression | Mental health services |
| 3. Chandrasekara (2016); Sri Lanka | Students N = 600 M age = not reported Range = 17 to 35 years 56% male | To examine the students’ attitudes toward seeking psychological help for mental health problems. | Cross-sectional | Mental health problems | Psychological help |
| 4. Cuyler (2019); USA | Military members N = 107 M age = 35 years Range = not reported 72% male | This paper investigates the mental health help-seeking intentions of military members using the TPB and the role of leadership support climate and co-worker support climate. | Cross-sectional | Mental health problems (namely anxiety and depression) | Mental health services |
| 5. Damghanian (2018); Iran | Community adults N = 1011 M age = 28 years Range = not reported 55% male | To use the TPB incorporated with self-stigma and perceived barriers to investigate the nature of help-seeking behaviors in a community sample at risk of anxiety or depression in Iran. | Cross-sectional | Mental health problems | Mental health services (psychologist, psychiatrist, psychiatry nurse, and general practitioner) |
| 6. Hammer (2018); USA | Community adults Study 1 N = 857 M age = 44 years Range = 18 to 85 years 30% male Study 2 N = 285 M age = 39.97 years Range = 18 to 76 years 20% male | To develop and examine the psychometric properties of the Mental Help Seeking Attitudes Scale (MHSAS). | Psychometric | Mental health concern | Mental health professional |
| 7. Hess (2013); USA | Students N = 889 M age = not reported Range = 18 to 58 years 38% male | To use the TPB to understand psychological help-seeking intention for three common concerns: Anxiety or Depression, Career Choice Concerns, and Alcohol or Drug Use. | Cross-sectional | Anxiety or depression, career choice concerns, and alcohol or drug use | Mental health professional |
| 8. Hyland (2015); Ireland | Police officers N = 331 M age = 28 years Range = 20 to 77 years 61% male | To investigate the construct validity, composite reliability and concurrent validity of the Inventory of attitudes towards seeking mental health services (IASMHS). | Psychometric | Psychological problems | Psychological counselling |
| 9. Hyland (2012a); Ireland 10. Hyland (2012b); Ireland | Emergency service workers N = 259 M age = 28 years Range = not reported 66% male | To examine intentions to engage in counselling among at-risk Irish government employees and to compare two alternative TPB models. | Cross-sectional | Psychological problems | Psychological counselling |
| 11. Karras (2018); USA | Veterans N = 809 M age = not reported Range = 44 to 65 years 84% male | Using the TPB as a guide, formative research was conducted to inform the design of suicide prevention messaging. | Cross-sectional | Suicidal thoughts and behaviors (STB) and mental health concerns | Treatment |
| 12. Kgathi (2011); Botswana | Students N = 236 M age = not reported Range = 16 to 41 years 100% male | To investigate predictors of men’s intentions to seek psychological help among a population of university students in Botswana | Cross-sectional | Psychological problems | Professional psychological help |
| 13. Lee (2016a); Canada | Military recruits N = 244 M age = 23 years Range = not reported 87% male | To identify personality and individual differences associated with mental health service use intentions within the TPB and explore relationships between these variables. | Cross-sectional | Mental health conditions | Mental health services |
| 14. Li (2017); China | Students N = 1128 M age = 20 years Range = 17 to 45 years 56% male | To investigate help-seeking behavior among Mainland Chinese college students by integrating the TPB and Andersen's Behavioral Model. | Cross-sectional | Personal or emotional problems | Professional psychological help / mental health services |
| 15. Li (2018); Australia | Students N = 611 M age = 21 years Range = not reported 34% male | To investigate correlates of Australian university students’ help-seeking intentions and actual service usage, testing and extending new models based on the TPB and the Behavioral Model of Health Services Use. | Cross-sectional | Mental health problem | Professional/psychological help |
| 16. Logsdon (2018a); USA | Latina immigrant mothers N = 50 M age = 28 years Range = not reported 0% male | To determine predictors of intention to seek depression treatment and to determine if intention to seek depression treatment differed in those with significant symptoms of depression. | Cross-sectional | Depression | Treatment |
| 17. Mackenzie (2004); Canada | Study 1 Community adults N = 208 M age = 46 years Range = 15 to 89 years 51% male Study 2 Students N = 297 M age = 21 years Range = not reported 49% male Study 3 Students N = 23 M age = 21 years Range = not reported 17% male | To adapt Fischer and Turner’s scale to address each of the conceptual and methodological limitations previously described. Our second goal is to extend the ATSPPHS to include new items according to the TPB with the aim of improving the inventory’s ability to predict mental health service use. | Psychometric | Psychological problems | Professional (psychologists, psychiatrists, social workers, and family physicians) |
| 18. Mak (2014); China (Macao) | Community adults Main study N = 337 M age = 34 years Range = 18 to 65 years 47% male | To compare the TPB and a partial mediation model of the TPB in explaining help-seeking intentions, and to examine the utility of the TPB in explaining intentions to seek mental health services in Macao. | Pilot study: questionnaire development Main study: cross-sectional | Symptoms such as depressed mood, anxiety, and sleep disturbance | Mental health services |
| 19. Mesidor (2014); USA | Students N = 111 M age = not reported Range = 18 to 36+ years 46% male | To investigate the extent to which attitudes, subjective norms, perceived behavioral control and psychological distress predict help-seeking intentions for international and African American college students. | Cross-sectional | Problems such as relationship difficulties, depression, personal concerns and drug-related problems | Mental health services / counselling |
| 20. Mo (2009); Hong Kong | Community adults N = 941 M age = 41 years Range = not reported 55% male | To apply the TPB to examine social cognitive correlates of intention to seek help for mental health problems among Chinese. | Cross-sectional | Mental health problems (e.g., low mood, feeling tense, anxious, problems in sleeping) | Mental health professionals (i.e., social workers, counsellors, clinical psychologists) |
| 21. Pumpuang (2018); Thailand | Nursing students N = 343 M age = not reported Range = not reported 2% male | To examine factors predicting nursing students’ intention to seek professional psychological help by using the TPB as a framework. | Cross-sectional | Psychological distress | Professional psychological help |
| 22. Schomerus (2009); Germany | Community adults Main study N = 2303 (2167 without depression, 136 with depression) M age = not reported Range = 18 to 60+ years 48% male | To determine what extent intentions to seek psychiatric help can be predicted by the TPB and compare models for people with and without depression. | Pilot study: qualitative preparation study Main study: cross-sectional | Depression | Psychiatric help |
| 23. Skogstad (2006); New Zealand | Prisoners Main study N = 527 M age = 30 years Range = 16 to 72 years 100% male | To assess whether prisoners' intentions to seek help for personal-emotional problems and suicidal feelings can be predicted using the TPB. | Pilot study: questionnaire development Main study: cross-sectional | Personal-emotional problems or suicidal feelings | Prison psychologist |
| 24. Stecker (2010); USA | Veterans N = 150 M age = 31 years Range = 20 to 59 years 88% male | To determine which beliefs predict the intention to engage and actual engagement in mental health treatment among veterans returning from Iraq. | Cross-sectional | Mental health concerns (targeted disorders include major depressive disorder, panic disorder, generalized anxiety disorder, PTSD and/or alcohol abuse disorder) | Mental health treatment (physician or mental health specialist) |
| 25. Teo (2020); China | Chinese students and their families Older generation: N = 275 M age = 40 Range = 30 to 53 years 51% male | This study applied an extended TPB model to investigate factors that contribute to the intention to use psychological cybercounseling in a Chinese sample. | Cross-sectional | Mental health problems | Cybercounseling (the practice of professional psychological counselling delivered by means of the internet or electronic technology) |
| 26. Tomczyk (2020); Germany | Community adults N = 188 M age = 50 years Range = not reported 29% male | To prospectively investigate help-seeking in a German community sample with currently untreated depressive symptoms. | Cross-sectional | Mental health problems | Mental health professionals (psychiatrist, psychotherapist, or clinical psychologist) |
| 27. Westerhof (2008); The Netherlands | Older adults N = 167 M age = 70 years Range = 65 to 75 years 48% male | To examine intentions to seek (preventive) psychological help among older persons. | Cross-sectional | Psychological problems (five common mental problems: depressive feelings, feelings of anxiety, loneliness, memory complaints, and sleep problems) | Professional psychological help (general practitioner, psychologist/psychiatrist) |
| 28. Zorrilla (2019); USA | Young community adults N = 430 M age = not reported Range = 18 to 24 years 39% male | To identify the predictors that influence help-seeking intentions on utilization of mental health services among a diverse population of young adults. | Cross-sectional | Depression and suicidal thoughts | Mental health professionals (social worker, counsellor/therapist, psychologist, or psychiatrist) |
| Theses |  |  |  |  |  |
| 1. Chang (2012); USA | Students N = 170 M age = not reported Range = 18 to 25 years 0% male | The current study uses the TPB as a model for changing attitudes toward seeking mental health help as well as increasing mental health counselling usage. | Baseline data from RCT | Depression | Counselling |
| 2. Clansy (1998); USA | African American parishioners Main study N = 352 M age = 39 years Range = 18 to 86 years 29% male | To examine the relationship between mental health ministries in African American churches and professional mental health help-seeking. | Pilot studies: questionnaire development Main study: cross-sectional | Common concerns (e.g., depression, anxiety, memory loss, marital trouble, drug, or alcohol abuse) | Professional mental health services |
| 3. Farmer (2013); UK | Dysphoric individuals Study 3 (i) N = 400 M age = 26 years Range = not reported 17% male | To examine whether the TPB and health belief model extended to incorporate identity as a depressed person was able to predict intentions to seek treatment, and current and prospective treatment seeking in depressed individuals. | Study 3: cross-sectional | Depression | Treatment |
| 4. Hamidi (2014); USA | International students N = 132 M age = 27 years Range = 18 to 40 years 54% male | The current study investigated Virginia Tech international students’ help-seeking process in terms of their attitudes toward counselling services, subjective norms, and perceived behavioral control. | Cross-sectional | Persistent issues (linguistic, academic, psychological, socializing, emotional, and personal issues) | Counselling services |
| 5. Hussain (2018); USA | Community adults N = 482 M age = 37 years Range = 18 to 70 years 55% male | This dissertation aims at the use of nostalgic emotional appeals to influence behavioral intentions to seek help. | Experimental between-group design | Depression | Professional counsellors |
| 6. Jarvis (2002); USA | Students N = 166 M age = 23 years Range = 17 to 50 years 33% male | This study examined the effects of attitudes, subjective social norms, and perceived behavioral control to predict help-seeking intentions of college students. | Cross-sectional | Psychological difficulties | Psychotherapy |
| 7. Lee (2016b); Canada | Second generation Chinese Canadian adults N = 212 M age = 27 years Range = 18 to 43 years 45% male | The present study examined the help-seeking process among second generation Chinese Canadians using the TPB. | Cross-sectional | Emotional problems | Mental health professional (including counsellor, psychologist, psychiatrist, other health care providers) |
| 8. Miller (2004); USA | Lawyers N = 405 M age = not reported Range = not reported 67% male | To investigate the attitudes and beliefs of lawyers towards seeking psychological services and identify determinants of intentions to seek help. | Cross-sectional | Psychological problem (anxiety, depression, severe stress, and drug and/or alcohol misuse) | Professional mental health treatment (individual and/or group therapy with a psychotherapist, psychologist, or psychiatrist) |
| 9. Mills (2010); USA | Students N = 143 M age = not reported Range = not reported 26% male | To relate disparate lines of research on suicide prevention and help-seeking using the TPB; to predict intentions to use campus mental health services. | Study 2: cross-sectional | Suicidality and emotional problems | Campus mental health services |
| 10. O’Flaherty (2017); UK | Community adults N = 236 M age = 41 years Range = 18 to 64 years 100% male | The current study aimed to examine factors that affect psychological help-seeking in men, using the TPB as a guide. | Chapter 2: cross-sectional | Mental health problems | Professional help-seeking sources (GP, mental health professional, and telephone helpline) |
| 11. Rathbone (2014); UK | Medical students N = 80 M age = not reported Range = not reported % male not reported | To use the TPB to understand psychological help-seeking intention for mental health problems in medical students. | Section B part two: cross-sectional | Mental health problems | Psychological help (mental health professional e.g., psychologist) |
| 12. Seyala (2011); USA | Older adults N = 401 (153 older adults, 248 baby boomers) M age = 61 years Range = 44 to 97 years 40% male | To determine whether baby boomers possessed similar or different intentions to seek mental health services compared to a current cohort of older adults. | Cross-sectional | Psychological problems | Mental health services (such as a psychologist, psychiatrist, counsellor, or social worker) |
| 13. Taylor (2018); Canada | Black Canadian adults N = 387 M age = 34 years Range = 18 to 80 years 17% male | To test the ability of the TPB model in explaining psychological help-seeking intention in a sample of 387 community-based Black Canadians. | Cross-sectional | Mental health problems (sad, depressed, anxious, worry) / personal or emotional problems | Mental health professionals (e.g., psychologists, psychiatrists, therapists, counsellors, and social workers) |
| 14. Walther (2002); Canada | Community adults N = 161 M age = 44 years Range = not reported 52% male | To gain a better understanding of which individuals are most likely to seek mental health services when they experience psychological problems. | Cross-sectional | Psychological problem | Mental health services (mental health professional, family physician) |
| 15. Woods (2013); USA | Black women N = 240 M age = 24 years Range = 18 to 60 years 0% male | To examine the relationship between the Strong Black Woman ideal, depressive symptoms, the TPB, and intentions to seek therapy. | Study 2: cross-sectional | Depression | Therapy |

| **Supplementary Table 2** Characteristics of studies - interventions | | | | | | | |
| --- | --- | --- | --- | --- | --- | --- | --- |
|  | | | | | | | |
| First author (year); country | Participants | Aim/purpose | Study design | Intervention type; description | Role of the TPB | Improved TPB variables | |
| Journal articles |  |  |  |  |  | |  |
| 1. Kauer (2017a)*; Australia 2. Kauer (2017b)**; Australia | Young community adults Kauer (2017a) N = 23 Kauer (2017b) N = 51 (Control group: 27, Intervention group: 24) | To describe the theoretical basis and explore the feasibility of the Link program, a world-first dedicated online navigation tool to facilitate mental health help-seeking for young adults. | Participatory design approach and pilot RCT | Online navigation tool: 'Link', a dedicated online mental health help-seeking tool that matches user’s mental health issues, severity, and service-type preferences (online, phone and face-to-face) with appropriate youth-friendly services. | The Link program is underpinned by the TPB and incorporates the Help-Seeking Model. Link aims to change attitudes by demonstrating the usefulness and ease of seeking help, improve subjective norms through decreasing stigma, and improving perceived behavioral control by increasing the accessibility of services and decreasing external barriers. | No (only intentions were assessed) | |
| 3. Logsdon (2018b); USA | Adolescent mothers N = 289 (Control group: 138, Intervention group: 151) | To test the effectiveness of an Internet-based depression intervention on seeking depression treatment. | Pre-post design | Internet-based depression intervention; the intervention was comprised of video vignettes of adolescent mothers describing their experiences with depression and how they sought treatment. Community resources e.g., referrals and hotlines, and answers to common questions about postpartum depression and mental health treatment were also included. | The components targeted attitudes, subjective norms, and perceived behavioral control. Video vignettes were designed to decrease stigma, increase normative beliefs, and increase attitudes. Resources were designed to increase perceived behavioral control. Common questions were designed to increase attitudes, decrease stigma, and increase self-efficacy. | Yes: attitudes, perceived behavioral control, intention and behavior. | |
| 4. Whealin (2014); USA | U.S. veterans N = 10 | To provide an overview of factors that affect symptomatic veterans’ decisions about whether to seek mental health services, to describe the TPB, and to describe the development of Considering Professional Help, a personalized web-based tool developed by the Department of Veterans Affairs. | Pilot pre-post design | Web-based psychoeducational tool; 'Considering Professional Help' was designed to encourage veterans to seek and engage in mental health care. It uses a tailored, interactive format to address key barriers, and users choose sections that address their unique concerns and perceived barriers or lack of efficacy. | The intervention is informed by motivational interviewing principles as well as cognitive–behavioral and social–cognitive theory. Based on the TPB and research to date, the intervention promotes attitude and behavior change by including credible sources and spokespersons who provide information to demystify the mental health treatment process. | Not assessed: improvements in willingness to consider treatment, stigma and barriers was observed. | |
| Theses |  |  |  |  |  |  | |
| 1. Chang (2012); USA | Female students N = 170 (Control group: 78, Intervention group: 92) | The current study uses the TPB as a model for changing attitudes towards seeking mental health help as well as increasing mental health counselling usage. | RCT | Experimental video; a 10-minute online film on going to a counselling center. Female actors talked about their experiences with depression and seeking help from the counselling center, that mimicked a personal disclosure to a webcam. | The intervention targeted three factors based on the TPB; a) personal attitudes toward counselling, b) perceived social norms about receiving counselling, and c) the perceived behavioral control of going to counselling. | Yes: attitudes, subjective norms, and perceived behavioral control immediately after intervention, subjective norms, and perceived behavioral control at one-month follow-up. | |
| 2. Hartong (2011); USA | Students N = 340 (Control group: 204, Intervention group: 136) | To test the impact of a video designed in line with the TPB on psychological help-seeking in college students. | Randomized post-test-only design | Experimental video: an 8-minute video designed to increase college students’ intentions to utilize mental health services when faced with a psychological problem. The video depicted the counselling process and included statements and testimonials. | Statements were written specifically with variables from the TPB in mind. | Yes: attitudes, subjective norms and perceived behavioral control. | |
| 3. Lindsley (2013); USA | Students N = 120 (Control group: 56, Intervention group: 64) | To determine the effectiveness of a multimedia intervention created to positively influence attitudes of university students towards seeking counselling. | RCT | Multimedia intervention; a 13-minute theory-guided presentation drawing on the TPB, information processing, and message effects theories. It included narration and pictures regarding treatment seeking for mental disorders. | All the content and language used in the intervention was designed to achieve optimal attitude change according to the relevant factors indicated by the TPB. | No† | |
| *Notes.* *Kauer, Buhagiar & Sanci (2017) **Kauer, Buhagiar, Blake, Cotton & Sanci (2017) †Taken from the results section not the abstract which reports different findings | | | | | |  | |
